# A Unique Mutation in a MYB Gene Cosegregates with the Nectarine Phenotype in Peach

**DOI:** 10.1371/journal.pone.0090574

**Published:** 2014-03-03

**Authors:** Elisa Vendramin, Giorgio Pea, Luca Dondini, Igor Pacheco, Maria Teresa Dettori, Laura Gazza, Simone Scalabrin, Francesco Strozzi, Stefano Tartarini, Daniele Bassi, Ignazio Verde, Laura Rossini

**Affiliations:** 1 Consiglio per la Ricerca e la Sperimentazione in Agricoltura – Centro di Ricerca per la Frutticoltura (CRA-FRU), Rome, Italy; 2 Parco Tecnologico Padano, Via Einstein, Loc. C.na Codazza, Lodi, Italy; 3 Università di Bologna, DipSA, Bologna, Italy; 4 Università degli Studi di Milano, DiSAA, Milan, Italy; 5 IGA Technology Services, Udine, Italy; Washington State University, United States of America

## Abstract

Nectarines play a key role in peach industry; the fuzzless skin has implications for consumer acceptance. The peach/nectarine (*G/g*) trait was described as monogenic and previously mapped on chromosome 5. Here, the position of the *G* locus was delimited within a 1.1 cM interval (635 kb) based on linkage analysis of an F_2_ progeny from the cross ‘Contender’ (C, peach) x ‘Ambra’ (A, nectarine). Careful inspection of the genes annotated in the corresponding genomic sequence (Peach v1.0), coupled with variant discovery, led to the identification of MYB gene *PpeMYB25* as a candidate for trichome formation on fruit skin. Analysis of genomic re-sequencing data from five peach/nectarine accessions pointed to the insertion of a LTR retroelement in exon 3 of the *PpeMYB25* gene as the cause of the recessive glabrous phenotype. A functional marker (indelG) developed on the LTR insertion cosegregated with the trait in the CxA F_2_ progeny and was validated on a broad panel of genotypes, including all known putative donors of the nectarine trait. This marker was shown to efficiently discriminate between peach and nectarine plants, indicating that a unique mutational event gave rise to the nectarine trait and providing a useful diagnostic tool for early seedling selection in peach breeding programs.

## Introduction

Peach (*Prunus persica* L. Batsch) is one of the most important fruit crops in temperate regions with about 21 million tons produced worldwide and Italy, with over 1.6 million tons, is the second producer after China (FAOSTAT 2011, http://faostat.fao.org/). The presence or absence of skin pubescence (fuzziness) is one of the commercial characteristics used to classify peach fruits along with flesh features (color, adhesion and texture) and fruit shape. Nectarines, characterized by the absence of fruit trichomes, are widely cultivated and play an important role in world peach production (30% in Italy, http://agri.istat.it/, 2013; 20% in USA, http://www.nass.usda.gov/, 2013) and may be associated with decreased allergenic properties. In *P. persica* two major allergens, Pru p 1 and Pru p 3, are known as responsible for the oral allergy syndrome (OAS) [1]. Indeed, the Pru p 3 protein was undetectable in the nectarine ‘Rita Star’ suggesting that this may be considered as a hypoallergenic variety [2]. Interestingly, in *Humulus lupulus* and in *Nicotiana tabacum*, genes encoding proteins highly similar to Pru p 1 and Pru p 3 are mainly expressed in trichomes [3], [4].

The peach/nectarine character is monogenic (*G/g*) with nectarine recessive to fuzzy fruit [5]. The *G* locus was mapped in the distal part of linkage group (LG) 5 [6], [7] spanning a region from 15,126,681 to 16,315,341 (1.189 Mb) of pseudomolecule 5 of the peach reference genome (Peach v1.0) [8]. Peach originated in North-West China and was domesticated there about 4,000–5,000 years ago [8]. From China it spread westwards reaching Persia following the Silk Road, was introduced to Rome in the first century BC and then disseminated to all the Roman Empire [9]. Nectarines have been known in China for over 2,000 years [10] and have been reported in most of the oases of the Tarim Basin (China) and along the Silk Road trade routes in Central Asia and the Caucasus [9], [11]. The means and timing of their introduction in Europe are not clear. Likely, Romans did not know this type of peaches [9], but nectarines have been described by several botanists in Europe since the Renaissance period [9]. Old European nectarine varieties include ‘Lord Napier’, ‘Precoce di Croncels’ and ‘Galopin’. In Southern Italy, traditional local white nectarines, called ‘Sbergie’ (Sicily) or ‘Merendelle’ (Calabria), have been cultivated since the 16^th^ century [12]. Cluster analysis suggested that these local accessions are distinct from the western nectarine germplasm, pointing to a putative different origin of this group of cultivars [13]. Historically, nectarines have had little impact in China's peach industry [11], and nowadays there are no reports of traditional nectarine cultivars available in China [14]. The timing of introduction of nectarines to the United States (US) is controversial: their cultivation is reported in the early 20^th^ century, although a newspaper article (New York Gazette March 28, 1768, p. 3) described nectarines being grown in the US prior to the War of Independence. Modern nectarine breeding started in the US in the middle of the 20^th^ century. In 1942, Anderson introduced the nectarine ‘Le Grand’ using the accession ‘Quetta’, discovered near the homonymous city in India (now part of Pakistan) in 1906 [15], as the source of the nectarine trait. Other known sources of the nectarine trait used in modern western breeding programs were ‘Goldmine’ and ‘Lippiatt’ discovered in New Zealand in 1900 and 1916, respectively [15]. These latter three genotypes are acknowledged as donors of most of the current nectarine cultivars widespread in US and Europe. Modern Japanese breeding programs have extensively used two old European nectarines, ‘Precoce di Croncels’ and ‘Lord Napier’, and modern US cultivars [16]. In the last decades, the trait was introduced to Chinese breeding programs directly from western accessions or indirectly using Japanese material [17].

Trichomes are hair-like appendages that derive from the differentiation of epidermal cells and are classified based on their morphology (unicellular or multicellular), and secretory abilities (glandular or non-glandular) [18], [19]. Trichomes may develop on several plant organs (leaf, fruit, seed, etc.). They play an important role in protecting plants against biotic and abiotic stresses [20]–[22] and can also hold a direct economic relevance. Aromatic substances are often synthesized by glandular trichomes, for example in aromatic plants, such as peppermint (*Mentha piperita*) [23] and basil (*Ocimum basilicum*) [24]. Cotton (*Gossipium hirsutum*) seed fibers are classified as non-glandular trichomes and represent one of the most highly expanded plant cell types [25]. In peach fruit, trichomes are non-glandular and unicellular and first appear on the ovary as early as four weeks before anthesis as observed in the peach ‘Contender’ [26]. By the time of physiological ripening most fruit skin trichomes are dead cells [27]. In *Arabidopsis* a number of genes involved in trichome formation and development have been identified by mutant analyses [28] and transcriptome profiling [29], revealing a complex regulatory network. Several transcription factors interact during trichome initiation and formation: in particular members of the R2R3-MYB class are known to act as positive regulators [30]–[33], while single-repeat MYB proteins function in negative control [34]–[36]. Mutations in the R2R3-MYB gene *GLABRA1* (*GL1*) result in glabrous plants in *A. thaliana* [37], *A. lyrata* [37], [38] and other Brassicaceae species [39]. In *Gossipium hirsutum*, *GhMYB25*, which encodes an R2R3-MYB factor, is involved in the differentiation of ovule epidermal cells into cotton fibers, as well as in the formation of leaf thricomes [40].

The aims of the present study were to precisely map the *G* locus, identify a candidate gene and develop a reliable marker for the nectarine phenotype (glabrous fruit). To these ends, we used an F_2_ population from a cross between the peach ‘Contender’ (C) and the nectarine ‘Ambra’ (A) [41], [42] to develop a Single Nucleotide Polymorphism (SNP) map around the *G* locus. Analysis of the corresponding region in the peach genome sequence (Peach v1.0) [8] led to the identification of an R2R3-MYB gene as a candidate for trichome formation in peach fruit. A functional marker (indelG) developed on this gene provides a useful tool for early seedling selection for the peach/nectarine trait in breeding programs.

## Materials and Methods

### Plant materials & DNA extraction

An F_2_ population of 305 seedlings derived from the cross between the peach ‘Contender’ (C) and the nectarine ‘Ambra’ (A), segregating for the peach/nectarine trait, [41], [42] (CxA F_2_), was used to develop a SNP map around the *G* locus. The trees were located in a farm belonging to the Municipality of Castel San Pietro (Bologna, Emilia Romagna, Italy) leased to ASTRA (latitude: from 44°24′44.18″N to: 44°24′30.08″N; longitude: from 11°35′47.21″E, to: 11°36′2.00″E). No specific permission was required because Daniele Bassi is the curator of the peach germplasm collection grown there and no endangered or protected species were involved.

Trees were planted on their own roots with a spacing of 1 m within and 4 m between rows and trained as slender spindle (one stem with short lateral scaffolds). Pruning was performed yearly and standard cultural practices were applied. Scoring of the peach/nectarine trait was carried out in two seasons to confirm correct scoring of the phenotype.

Ninety-five *P. persica* genotypes, 46 peaches and 49 nectarines grown at the CRA-FRU experimental farm (Rome, Italy) (except for ‘Galopin’ and ‘Lord Napier’ grown at Ivalsa-CNR, Follonica, Italy), were analyzed to validate the functional marker. For each accession, phenotype, pedigree, geographical origin and putative donor of the nectarine trait are reported in Table 1. DNA was extracted from leaf tissue using the DNeasy Plant Mini Kit (Qiagen GmbH, Hilden, Germany) as per manufacturer's protocol and quantified with NanoDrop (Thermo Scientific, Waltham, MA, USA).

**Table 1:** List of 95 peach/nectarine varieties representing the diversity of cultivated *P. persica*.

Accession name	Genotype	Phenotype	Pedigree	Geographical origin^a^	Putative donor of nectarine allele^b^
Ambra	197-197	nectarine	Stark Red Gold x Mayfire	Italy (B)	Lippiatt/Goldmine
Aniversario	197-197	nectarine	–	Argentina (B)	-/-
Big Top	197-197	nectarine	–	USA (B)	-/-
Branca	197-197	nectarine	Goldmine x Pala	Brazil (B)	Goldmine/Goldmine
California	197-197	nectarine	P60-30 x Fantasia	Italy, 1994 (B)	Quetta/-
Centenaria	197-197	nectarine	Docura 2 op	Brazil (B)	Goldmine/Goldmine
Chiyodared	197-197	nectarine	Hirakutsared x Nectared 5	Japan (B)	Lord Napier or Precoce di Croncels/-
Crasiommolo Rosso	197-197	nectarine	–	Italy (L)	-/-
Crimson Gold	197-197	nectarine	Nectarine selection x July Elberta	USA, 1967 (B)	-/-
Sabrina	197-197	nectarine	–	Spain (B)	-/-
Fairlane	197-197	nectarine	(Le Grand x Sun Grand) x Fantasia	USA, 1973 (B)	Lippiatt/Quetta
Fantasia	197-197	nectarine	Gold king x p101-24 ( = Red King op)	USA, 1969 (B)	Quetta/Quetta
Firebrite	197-197	nectarine	Flavortop x (Red King F_2_)	USA, 1974 (B)	Quetta/-
Fuzador	197-197	nectarine	((Greensboro op x Elberta) x JHHale) F_2_	France, 1973 (B)	-/-
Galopin	197-197	nectarine	–	England, 1869 (L)	-/-
Gold Mine	197-197	nectarine	–	New Zeland, 1900 (B)	
Golden Grand	197-197	nectarine	Le Grand x (Le Grand x Kim)	USA, 1954 (B)	Quetta/Lippiatt
Jacquotte	197-197	nectarine	–	France, 1979 (B)	-/-
Laura	197-197	nectarine	–	USA, 1995 (B)	
JinXia	197-197	nectarine	(Okubo x Okitsu) x Okitsu F_2_	China (B)	Lord Napier/Precoce di Croncels
Lord Napier	197-197	nectarine	–	Belgium, 1859 (L)	-/-
Madonna Di Agosto	197-197	nectarine	–	Italy (L)	-/-
Madonna Di Giugno	197-197	nectarine	–	Italy (L)	-/-
Magali	197-197	nectarine	–	France, 1988 (B)	-/-
Max	197-197	nectarine	–	Italy, 1995 (B)	-/-
Mayfire	197-197	nectarine	Armking op	USA, 1984 (B)	Goldmine/Goldmine
Nectaross	197-197	nectarine	Stark Red Gold x Le Grand	Italy, 1983 (B)	Lippiatt/Quetta
Nettarina Pendula	197-197	nectarine	–	Italy (L)	-/-
Nico	197-197	nectarine	–	Italy (B)	-/-
Phn 91-12	197-197	nectarine	–	USA (B)	-/-
Phn 91-14	197-197	nectarine	–	USA (B)	-/-
Phn 91-17	197-197	nectarine	–	USA (B)	-/-
Quetta	197-197	nectarine	–	Pakistan, 1906 (L)	
Ricci 2	197-197	nectarine	Stark Red Gold x Tastyfree	Italy, 1993 (B)	Lippiatt/Quetta
Romamer 1	197-197	nectarine	–	Romania, 1983 (B)	-/-
Romamer 2	197-197	nectarine	City 29-245 x RR48-153	Romania, 1983 (B)	-/-
Russian Nectarine 592-81	197-197	nectarine	–	Ukrayne, 1980 (L)	-/-
Russian Nectarine 598-81	197-197	nectarine	–	Ukrayne, 1980 (L)	-/-
Shizukured	197-197	nectarine	Okitsu x NJN17	Japan (B)	Lord Napier or Precoce di Croncels/-
Silver Lode	197-197	nectarine	(Goldmine x Rio Oso Gem) x (Goldmine x July Elberta)	USA, 1951 (B)	Goldmine/Goldmine
Sirio	197-197	nectarine	Flamekist x Fantasia	Italy, 1987 (B)	Quetta/Quetta
Snow Queen	197-197	nectarine	–	USA, 1975	-/-
Souvenir Nikitski	197-197	nectarine	Lola op	Ukrayne, 1988 (B)	-/-
StarkRedgold	197-197	nectarine	Sun Grand op	USA (B)	Lippiatt/Lippiatt
Summer Beauty	197-197	nectarine	Red Diamond x Sun Grand	USA, 1979 (B)	Quetta/Lippiatt
Vania	197-197	nectarine	–	Italy, 1990 (B)	-/-
ZeeGlo	197-197	nectarine	(Red Grand op) x (Sun Grand x Merril Gem)	USA, 1988 (B)	Quetta/Lippiatt
Zephyr	197-197	nectarine	–	France, 1992 (B)	-/-
Zincal 5	197-197	nectarine	–	Spain (B)	-/-
Acireale	941-941	peach	–	Italy (L)	
Amber Gold	941-941	peach	Red Grand x Royal May	USA, 1973 (B)	
Amsden	941-941	peach		USA, 1868 (B)	
Autumnglo	941-197	peach	(Candoka x Tennessee Natural) x Merril Fiesta	USA, 1977 (B)	-
BaekmiJosaeng	941-941	peach	Mishima x Nunome Wase	Republic of Korea, 1983 (B)	
Baldagenais	941-197	peach	–	France	-
Changbang Mutant	941-941	peach	–	Republic of Korea, 1986 (B)	
Chiyomaru	941-941	peach	–	Japan, 1988 (B)	
Chui Huang Tao	941-941	peach	–	China (B)	
Ciccio Petrino	941-941	peach	–	Italy	
City 32-82	941-197	peach	–	USA (B)	-
Contender	941-941	peach	Wiblo x [Norman x (Candor x (Summercrest x Redhaven))]	USA, 1987 (B)	
Cp 88/2	941-941	peach	–	Mexico (B)	
Early Gold	941-941	peach	–	Japan, 1980 (B)	
Elberta	941-941	peach	Chinese Cling op (perhaps x Early Crawford)	USA, 1889 (B)	
CxA F_1_	941-197	peach	Contender x Ambra	Italy (B)	Lippiatt or Goldmine
Fairtime	941-197	peach	(Rodeo x Kirkman Gem) op	USA, 1968 (B)	
Fayette	941-941	peach	Fay Elberta x (Fireglow x (Fireglow x Hiley))	USA, 1966 (B)	
FeiChing Bai Li 17	941-941	peach	–	China (B)	
FeiChing Tao	941-941	peach	–	China, 1909 (B)	
Fidelia	941-197	peach	(O'Henry x Giant Babcock) x (May Grand x Sam Houston)	USA, 1986 (B)	-
Frau Maria Rudolf	941-941	peach	Top Red Delicious op	Germany (B)	
Grosse Mignonne	941-941	peach	–	France, 1667 (L)	
Higama	941-941	peach	Japanese seed op	France, 1970 (B)	
Hwando 1	941-941	peach	–	Republic of Korea, 1977 (B)	
J H Hale	941-941	peach	putative selfpollination of Elberta	USA (B)	
JingYu	941-197	peach	Okubo x Okitsu	China (B)	Lord Napier or Precoce di Croncels
KurakataWase	941-941	peach	–	Japan (B)	
PLove2- 2N	941-941	peach	di-haploids of Lovell	USA, 1882 (B)	
O'Henry	941-197	peach	Merrill Bonanza op	USA, 1970 (B)	-
Pantao 20-58	941-941	peach	–	China, 1869 (B)	
Pillar	941-941	peach	–	Japan (B)	
Redhaven	941-941	peach	Halehaven x Kalhaven	USA, 1940 (B)	
Reginella II	941-941	peach	–	Italy (B)	
Rou Tao	941-941	peach	–	China (L)	
Russotto	941-941	peach	–	Italy (B)	
Sahua Hong Pantao	941-941	peach	–	China (L)	
Sanguinella	941-941	peach	–	Italy (L)	
Shen Zhou Mi Tao	941-941	peach	–	China (B)	
Siberian C	941-941	peach	selection of a China seedling	Canada, 1967 (B)	
Summer Pearl	941-197	peach	[(Candoka x Tennessee natural op) x ((Candoka x Flaming Gold) x NJN5 op)] x (PI119844 x White Hale)	USA, 1979 (B)	-
TaturaDawn	941-941	peach	Levis selfpollination	Australia, 1960 (B)	
Yoshihime	941-197	peach	(Nakatsu Hakuto x Nunome Wase) x Akatsuki	Japan, 1990 (B)	-
Yu Bai	941-941	peach	–	China (L)	
Yumyeong	941-941	peach	Yamato-Wase x Nunome Wase	Republic of Korea, 1977 (B)	
ZansetsuShidare	941-941	peach	–	Japan (B)	

a (B)  =  breeding materials (L)  =  landrace.

b -  =  unknown donor of the nectarine allele.

For each accession the following information is reported: name, genotype at the indelG marker (941 bp reference peach allele, 197 bp nectarine allele carrying the retrotransposon insertion), phenotype, pedigree, country of origin and year of release/discovery when known, putative donor of the nectarine allele in *P.persica* if pedrigree information was available.

### Linkage map

In total 305 individuals from the CxA F_2_ progeny were analyzed to map the *G* locus using an upgraded version of the CxA map [41], [42] covering LG 5 from base 10,192,138 to base 17,544,073 of the peach genome sequence pseudomolecule 5 and spanning the already known *G* interval [7]. To refine the position of the *G* locus, SNPs located in this region were selected from those identified through analysis of re-sequencing data from the CxA F_1_ individual [42] (biosample SRS335631, run SRR502997). About 300 bp of the SNP-flanking sequence were downloaded from the IGA peach Gbrowse (http://www.appliedgenomics.org/) and the Mass ARRAY Assay Design 3.1 software was used to design multiplex assays for SNP analysis [43]. SNP genotyping was performed using the iPLEX Gold technology available for Sequenom platforms (Sequenom, Inc., San Diego, CA, USA). SNP markers and the *G* locus (scored as a dominant phenotypic marker) were mapped using Joinmap 3.0 [44] with a minimum LOD score of 10 for grouping; the Kosambi mapping function [45] was used to convert recombination frequencies into map distances. Based on this map, three new SNP markers (S5_15988499, S5_15865556 and S5_15866258) were developed in the *G* locus interval (scaffold_5, from 15,853,006 to 16,488,104; see Results and Discussion) and genotyped on eight informative recombinants by sequencing 200 bp encompassing the SNP (primer sequences shown in Table 2). Standard PCRs were performed using GoTaq Green Master Mix (Promega, Madison, WI, USA). Each PCR reaction contained 1 X GoTaq Green Master Mix, 0.4 µM of each primer, 20 ng template DNA and sterile Milli-Q water to a final volume of 25 µl. The PCR protocol consisted in an initial step at 95°C (5 min), followed by 40 cycles at 95°C (30 s), 60°C (30 s) and 72°C (1 min), and a final elongation at 72°C (5 min). PCR products were purified with ExoSapIT (Amersham PharmaciaBiotech, Uppsala, Sweden) and sequenced with the Big Dye Terminator v3.1 Cycle Sequencing Kit (Applied Biosystems, Foster City, CA, USA). After ethanol precipitation, sequencing products were mixed with 15 µl of HiDi formamide and subjected to capillary electrophoresis in an ABI Prism 3730 DNA Analyzer (Applied Biosystems, Foster City, CA,USA). Genotyping was performed by visual inspection of the resulting electropherograms using 4PEAKS freeware (Nucleobytes Inc.).

**Table 2:** SNP markers.

Markers^a^	cM^b^	Peach v1.0 position (nt)	SNP allele^c^	Forward primer	Reverse primer	SNP Extension primer d
S5_10192138	0	10,192,138	G/A	GATGAATGGGTGAAGGTAAG	TTCCGCAAAAAAAAACATATC	ttaagGGGTGAAGGTAAGTTTGCACA
S5_11640083	4.2	11,640,083	T/G	CCTACTACACAATTGCCTTA	GTCGGTCGTCAGTTTTTTTG	cgacCTTGTAGATTCTAATGGAAGTA
S5_12847567	11.4	12,847,567	T/A	TGCGGATTTTTCTTAGCTAC	CTCTTTCCCAATCTCAATCG	AATCGCATTGTTGAGAC
S5_13449464	14.3	13,449,464	G/C	CGGTGATTGATATGATGATG	CCACTCAAATTGCCTTTCCC	GTGATTGATATGATGATGATTTATAT
S5_13852617	16.7	13,852,617	A/C	ATACCTATGTTCACTCCCCG	TTGGCTGGTAAGGTTGTTGG	TGTACTGATGTGTGAATCTAATG
S5_14584095	24.3	14,584,095	G/C	AAGTTGTTCCAGTGGCAACC	TATAGTGGGGCTGGAATCTG	cccGGCTGGAATCTGTTCTCTCAGAC
S5_14894563	25.5	14,894,563	C/A	GCAGAGGAATTTTTCCCTAC	TTAGGGAGGGAGCTATGTTG	gGTTGGAGGTATTTGGGC
S5_14949332	26.0	14,949,332	T/C	AGAAGATGTGGTTCCAGAGG	TTCTCGATCCGGAAGGAGAT	TCCCAGATCCAAGACCC
S5_15731107	28.4	15,731,107	T/G	CTGTTGTAAGACAGGTTTGG	AACATGCTTGCGGCTTCGTC	GGCTTCGTCCATACGCC
S5_15853006	28.8	15,853,006	T/A	TAGTTTGTCTGTCAAACCGC	CCGAGAAGACTGAAGAGTTG	GCTATTAAAGACATTAGAGATGA
*S5_15865556*	*29.1*	15,865,556	A/T	GGTTGGGGCCTCTGTATTCT	TAAAGGCAACCACATTGCAG	
*S5_15866258*	*29.1*	15,866,258	A/G	TCAGCTTGTCCATGGCATTA	GCCGTAAAGGCTTTCCTCTC	
*S5_15988499*	*29.4*	15,988,499	C/T	GCCGTGAAGTGGAGTTCTCT	GATTCTCACTCTGCTCCTGTCT	
S5_16488104	29.9	16,488,104	A/G	TTCGCATTCATTAGTTCAC	CAGGTTTGTGAGTTTGCTTG	TACTAAACGGAAGCTATGT
S5_17544073	36.2	17,544,073	G/A	GCCATCTCTCTGTTTCTCTG	GTAGTATCAGCCGACTGTAG	cTAACATACATGACATGACATACACCC

a SNP detection was performed by Sequenom MassArray technology, except for the SNPs in italics that were genotyped by Sanger sequence.

b the genetic distances estimated by the analysis of informative recombinant plants are reported in italics.

c the first nucleotide correspond to the reference allele in the peach genome (Peach v1.0).

d lower case bases correspond to the tails added to the SNP extension primer for Sequenom MassArray analysis.

**Figure 1:**
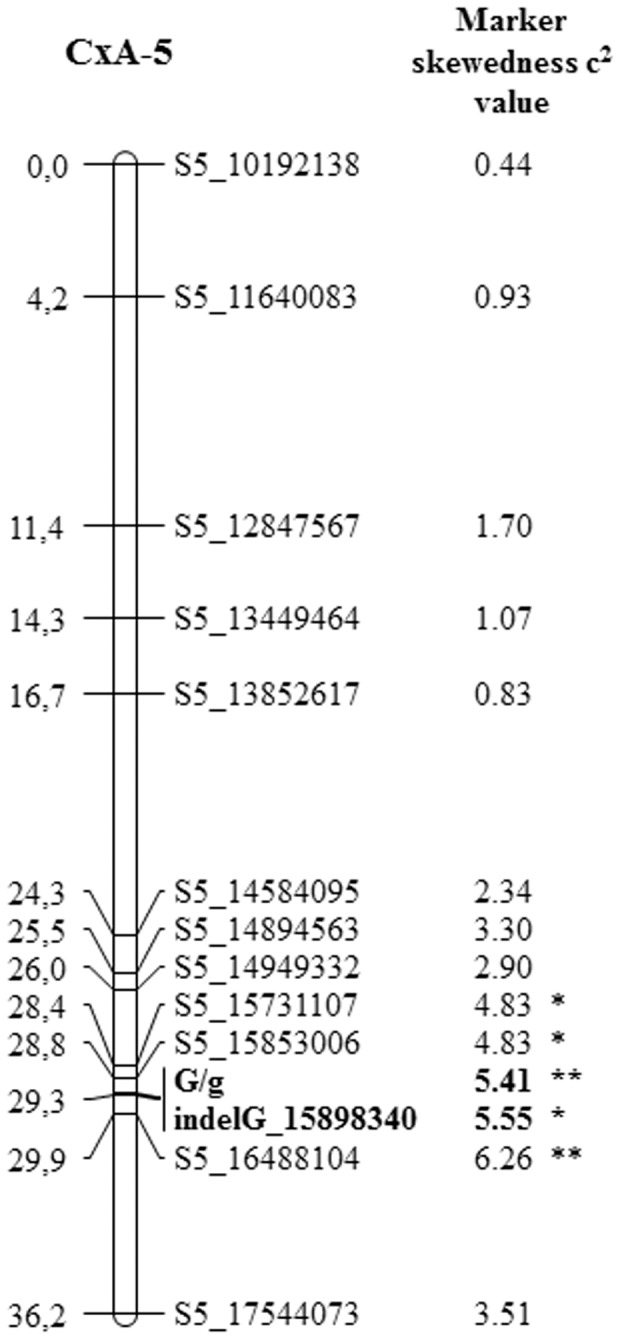
LG 5 CxA map around the *G* locus. Linkage map obtained from analysis of the CxA F_2_ progeny. On the left side distances are indicated in cM; on the right the marker name, the physical position on Peach v1.0 and marker skewedness are reported. The peach/nectarine locus and the indelG marker are shown in bold.

For each marker the following information is reported: marker name, position in cM with respect to the map in Figure 1, position in bp with respect to the peach genome sequence (Peach v1.0), SNP allele and the primer sequences used.

### Variant discovery from NGS data

In order to identify genetic variants putatively involved in the control of the nectarine trait publicly available paired-end (PE) whole-genome re-sequencing data of *P. persica* accessions from study SRP013437 [8] were downloaded from the NCBI Sequence Read Archive (SRA) [46]. Five accessions were considered for this study: ‘Bolero’ (biosample SRS335629, run SRR501836), ‘OroA’ (biosample SRS335635, run SRR502986), ‘Lovell’ Clone PLov2-2N (biosample SRS335634, run SRR502985), ‘Quetta’ (biosample SRS335636, runs SRR502989 and SRR502987) and F_1_ ‘Contender’ × ‘Ambra’ (biosample SRS335631, run SRR502997). ‘Quetta’ was included as the reference nectarine accession. The CxA F_1_ individual originated the CxA F_2_ population used to map the *G* locus. The peach ‘Lovell’ Clone PLov2-2N is the doubled haploid used to generate the reference peach genome sequence (Peach v1.0) [8], providing an internal control for false variant calling. Finally, the peaches ‘Bolero’ and ‘OroA’ were chosen as controls for nectarine segregation, as it has been demonstrated that the nectarine trait does not segregate in the ‘Bolero’ × ‘OroA’ F_1_ population [42].

SRA data of each run were dumped in fastq format using the *fastq-dump* tool of NCBI sratoolkit v2.1.16 software (http://www.ncbi.nlm.nih.gov/Traces/sra/sra.cgi?view=software), splitting forward and reverse paired reads for each sample into two separate files. Reads were quality filtered on a single sample basis using Trimmomatic v0.22 [47], first trimming leading and trailing bases below a quality threshold of 20, and then removing trimmed reads shorter than 24 bp or having an average quality below 20 (calculated on 8 bp long sliding windows). For each sample, only reads passing the quality filtering as matching pairs were retained and aligned to the whole *P. persica* reference genome Peach v1.0 using the Burrows-Wheeler Alignment Tool (BWA v0.6.2) [48]. The *aln* (IS linear-time algorithm) and *sampe* (all default options except *-n* 25 *–N* 25) commands were applied, respectively, for finding suffix array (SA) coordinates of each individual read and to convert them to chromosomal coordinates and to pair the reads. The resulting alignment SAM files were converted by Picard Tools version 1.77 (http://picard.sourceforge.net/) to sorted BAM files compliant to the Genome Analysis Toolkit (GATK) format, using the tools *CleanSam*, *SamFormatConverter* and *AddOrReplaceReadGroups*. GATK-compliant BAM files were submitted to GATK version 2.3–3 [49] for pre-processing procedures, i.e. indel realignment, duplicate removal and base quality score recalibration (BQSR). The data table needed for the recalibration step in BQSR was manually generated upon validated SNP data from the Peach 9K chip array [50]. Variant discovery procedures were then applied using whole-genome recalibrated alignments of all five samples simultaneously. Genotypes for SNP and small INDEL variants were called through the GATK *HaplotypeCaller* tool applying hard filtering parameters [51]. Structural variants were also independently called on the same recalibrated alignment data by Pindel software v0.2.4t [52] following standard procedures.

Reads from the resequencing of ‘Quetta’ (biosample SRS335636, runs SRR502989 and SRR502987) were also analyzed using the CLC genomic workbench (6.0.1). The 75 bp pair-end fragments were trimmed for quality retaining only nucleotides with Phred values higher than 30, and trimmed reads were aligned against the *P. persica* reference genome (Peach v1.0) [8] using the read mapping tool. Only reads with over 90% identity over at least 92% of their length were mapped on the reference. All variant discovery searches were limited to the locus *G* mapping interval defined by informative recombinants in the C×A F_2_ mapping population (scaffold_5, from 15,853,006 to 16,488,104, see Results and Discussion).

### Validation of candidate variant

To validate the putative variant individuated among the resequenced genotypes long-range PCRs were performed on five nectarines genotypes (‘Quetta’, ‘Goldmine’, ‘Madonna di Agosto’, ‘Stark Red Gold’ and ‘Ambra’) and on the peach ‘Contender’, with primers Seq16F and Seq4R (Table 3) designed flanking the putative insertion, using Herculase DNA Polymerase (Agilent Technologies, Santa Clara, CA, USA). Each reaction contained 1x Herculase reaction buffer, 0.3 mM dNTP mix, 0.5 µM each Seq16F and Seq4R primer, 3% DMSO, 2.5 U Herculase polymerase, 300 ng template DNA, and sterile Milli-Q water to a final volume of 50 µl. The following PCR protocol was performed on a Esco Swift Maxi thermocycler (Esco GB Ltd, Downton, UK) or an Applied Biosystems 2720 Thermal Cycler (Applied Biosystems, Foster City, CA, USA): 95°C for 5 min; 28 cycles of 95°C (1 min), 59°C (1 min), 72°C (12 min) followed by a step at 72°C for additional 12 min. All PCR amplicons were checked on 1% agarose gel in an overnight run in SB buffer. A standard ethidium bromide staining was used for band visualization.

**Table 3:** List of Primers.

Name	Sequence 5′->3′	Start Position on Scaffold 5 (nt)	Len	Notes
Seq16F	ATTCCGCTCCTCATGTAGTACA	15,898,340	22	upstream the LTR insertion
Seq4R	CCAAATAAACCACCACCTACTCTGTTA	15,899,297	27	downstream the LTR insertion
Seq15F	GCTGTAGGCTAAGGTGGAGAA	15,898,078	21	RT-PCR primer designed on exon 2 of *PpeMYB25*
Seq15R	ACTGAGCAATGTGGCTGAGA	15,898,586	20	RT-PCR primer designed on exon 3 of *PpeMYB25*
ppaRPII_RT-F	TGAAGCATACACCTATGATGATGAAG		26	RT-PCR housekeeping, [53](Tong et al, 2009)
ppaRPII_RT-R	CTTTGACAGCACCAGTAGATTCC		23	RT-PCR housekeeping, [53](Tong et al, 2009)
indelG-F	CTTGCACCTGAGTTCGATTCCG	15,898,324	22	upstream the indel in *PpeMYB25*
indelG-1R	GGCTTCAATGGCAGAACAAGG		21	within left border of the indel in *PpeMYB25*
indelG-2R	GCAGGTGGTGGAGATTCATTCAT	15,899,264	23	downstream the indel in *PpeMYB25*

Name, sequence, position on Peach v1.0 and length of primers used to perform long-range PCR, RT-PCR and indelG assay.

For restriction analysis, 5 µl of long-range PCR products from ‘Quetta’, ‘Goldmine’, ‘Madonna di Agosto’, ‘Stark Red Gold’ and ‘Ambra’ were digested with *EcoR*I and *Hind*III (Fermentas, Vilnius, Lithuania) in a single overnight reaction at 37°C. Master mix was calculated by double digest tool (http://www.thermoscientificbio.com/webtools/doubledigest/) with 0.5 U/sample of each restriction enzyme and buffer R. Restriction products were separated on 1% agarose gels and stained with ethidium bromide for band visualization.

The ‘Quetta’ long-range PCR product was first purified with the Macherey-Nagel PCR clean-up kit (Carlo Erba reagents, Italy) and quantified by Picogreen (Quant-iT PicoGreen dsDNA kit, Life Technology, US) in preparation for sequencing using the Illumina MiSeq platform with a 150 bp paired end sequencing strategy. Preparation of Nextera XT library was performed with 1 ng of genomic DNA according to the Nextera XT protocol (Ver. Oct 2012, rev C). Briefly, the DNA was fragmented in 5 µl of Amplicon Tagment Mix and 10 µl of Tagment DNA buffer (Illumina, San Diego, CA, USA). Tagmentation reactions were performed by incubation at 55°C for 5 min followed by neutralization with 5 µl of Neutralize Tagment Buffer for 5 min. Tagmented DNA (25 µl) was used as the template in a 50 µl limited-cycle PCR (12 cycles) and processed as outlined in the Nextera XT protocol. Amplified DNA was purified using 90 µl of AMPure XP beads then normalized with 45 µl of combined Library Normalization beads/additives. In preparation for cluster generation and sequencing, the normalized library was diluted in hybridization buffer and heat denatured. Due to the low diversity of the library a phiX spike-in (30%) was added to the final denatured 10 pM library. The sample was sequenced using the MiSeq Personal Sequencer (Illumina Inc., San Diego, CA, USA) running MiSeq Control Software Version 2.0.

A total of 20 M reads were obtained and analyzed using the CLC genomic workbench (6.0.1). Trimming and De Novo assembly tools were used with default parameters. The assembly obtained was filtered retaining only contigs with a length greater than 200 bp and formed by more than 500 reads. The filtered contigs were mapped using BWA v0.7.5a [53] with the MEM algorithm against the Peach v1.0 genome. Identification of conserved domains on contigs was performed using HMMER v3.1 [54] against the PFAM [55] database v27.0. Blast search of contigs was done using NCBI-BLAST+v2.2.27 [56] against the NT database downloaded from NCBI FTP site on Oct 23 2013.

### Expression analysis

Total RNA was extracted from floral buds collected from ‘Contender’ and ‘Ambra’ at different developmental stages (seven, five, four and one week before anthesis) using the RNeasy Plant Mini Kit (Qiagen GmbH, Hilden, Germany) and treated with DNAse I (Sigma-Aldrich, St. Louis, MI, USA) following manufacturers' instructions; 1 µg of RNA was reverse-transcribed using the GoScript Reverse Transcription System (Promega, Fitchburg, WI, USA) with oligo (dT)_15_ according to the manufacturer's protocol. For reverse transcription analysis, primers were designed on exon 2 (Seq15F) and exon 3 (Seq15R) of the *PpeMYB25* gene and the RNA polymerase II sequence was used as reference gene [57] (Table 3). For RT-PCR, 1 µL of cDNA was used with 1x GoTaq Green Master Mix, 0.4 µM of each primer and sterile Milli-Q water to a final volume of 10 µl. The PCR protocol consisted of an initial denaturation at 95°C for 2 min, followed by 35 cycles at 95°C (20 s), 62°C (20 s) and 72°C (30 s), followed by a final elongation at 72°C (5 min). PCR products were checked on 1% agarose gel at 5 V/cm in TBE buffer. A standard ethidium bromide staining was used for band visualization.

### Functional marker design and genotyping

To perform association studies for the nectarine trait and provide a tool for marker assisted breeding (MAB) a codominant marker (indelG), consisting of a three-primer PCR assay (primers indelG-F, indelG-1R and indelG-2R; Table 3), was developed for genotyping of the candidate insertion: two outer primers (one forward and one reverse), designed on opposite sides of the insertion were combined in a single reaction with an inner reverse primer (designed on the reconstructed left sequence of the insertion).

PCRs were carried out in 10 µl containing 10 ng of template DNA, 1x PCR buffer, 1.5 mm MgCl_2_, 200 µM each dNTP, 0.2 µM each primers and 0.5 U of Platinum Taq DNA polymerase (Life Technologies, Carlsbad, CA, USA). Amplifications were performed on a Veriti thermal cycle (Life Technologies, Carlsbad, CA, USA) with the following temperature profile: 95°C (5 min) followed by 35 cycles at 94°C (30 s), 61°C (30 s), 72°C (30 s) and a final extension at 72°C (10 min). PCR products were separated on an ethidium bromide stained 1% agarose gel. The 305 seedlings of the CxA F_2_ population, the parents and the hybrid CxA F_1_, as well as 46 peach and 49 nectarine accessions, were analyzed (Table 1).

## Results and Discussion

### Mapping of the G locus

Scoring of the CxA F_2_ progeny revealed the presence of 246 peach and 59 nectarine plants, indicating a slight distortion from the expected 3∶1 segregation (χ^2^ = 5.41, *p*<0.05 with 1 d.f.). In agreement with a previous report [7], the peach/nectarine phenotype was regarded as a dominant trait (*G* locus). A total of 12 SNPs, covering about 7 Mb of LG 5 around the *G* locus, were mapped on the whole progeny (Figure 1). Consistent with the observed distortion of the phenotypic trait, a skewed segregation was also found for all the markers around the *G* locus from S5_15731107 to S5_16488104 (Figure 1). The *G* locus was placed between markers S5_15853006 and S5_16488104 within an interval 1.1 cM (635 kb, Figure 1). Three additional SNPs inside the *G* region (S5_15865556, S5_15866258 and S5_15988499) were also successfully mapped by genotyping of informative recombinant plants. However none of them were useful to refine the position of *G* locus (data not shown).

### Variant identification in the locus G genomic region

**Figure 2:**
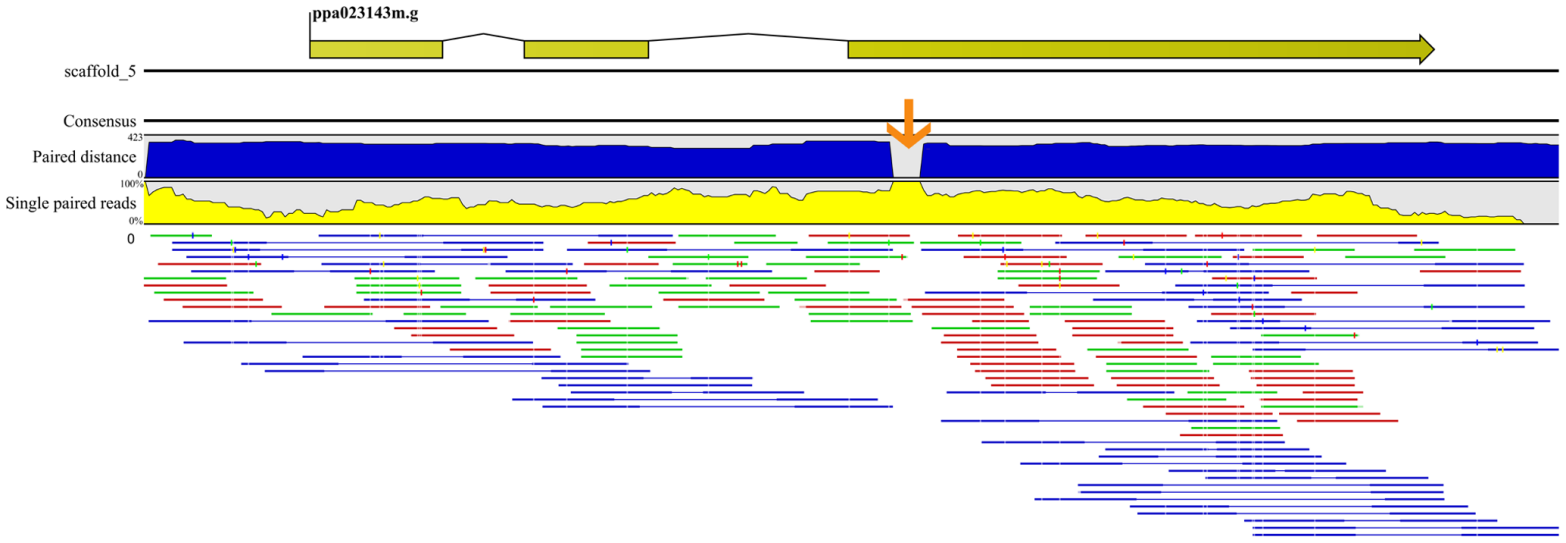
Alignment of Quetta reads against a 635 kb interval of Peach v1.0 pseudomolecule 5. Alignment results of reads, obtained by the resequencing of ‘Quetta’, against the peach genome region identified by the mapping interval in LG5 (from 15,853,006 bp to 16,488,104 bp). Top panel: intron-exon structure of *ppa023143m*. Central panel: plot of ‘Quetta’ paired-end distance (blue) and frequencies of single reads (yellow) at the *ppa023143m* locus. Bottom panel: blue lines are paired reads, green and red lines correspond to single reads with missing mate on the right and left side, respectively. The orange arrow points to the putative insertion inside exon 3 of *ppa023143m*.

In order to identify variants putatively underlying the nectarine phenotype, genome-wide recalibrated alignment data from five *P. persica* accessions were examined in detail around the *G* locus (scaffold_5, from 15,853,006 to 16,488,104). In this region, GATK *HaplotypeCaller* detected 291 SNP and indel variants above the chosen minimum phred-scaled score quality threshold of 200, out of which 67 mapped within predicted genes in the peach genome (Peach v1.0) [8]. Of the latter, 20 variants, distributed in seven genes, were heterozygous in CxA F_1_ with non-segregating joint genotype combinations in ‘Bolero’ and ‘OroA’. In the nectarine ‘Quetta’ the same analysis identified two variants, both indels, homozygous for the non-reference allele and located in two distinct genes (*ppa023682m* and *ppa023143m*). Both of them were heterozygous in CxA F_1_, where no other variants were found in these two genes, and were thus considered as putative candidates for the *G* locus. The first candidate variant is a deletion of 3 motives in a known (AAAC)_6_ microsatellite at position 16,463,040 which maps within the only intron of the predicted gene *ppa023628m* (best *Arabidopsis thaliana* blastx match AT3G29575.1, ABI five binding protein *AFP3*, e-value 5×10^−18^). ABI Five Binding Proteins (AFPs) are members of a small plant-specific protein family, characterized by three conserved domains of unknown function. AFPs act as negative regulators of ABA signaling [58] and have no known involvement in trichome formation. Next we focused on the second variant, reported by *Haplotype Caller* as two distinct insertions of 76 and 49 bp on the left and right side, respectively, of an (AC)_3_ motif at position 15,898,458. This INDEL variant maps to the last exon (exon 3) of the predicted gene *ppa023143m* (best *Arabidopsis thaliana* blastx match AT5G15310.1, MYB domain protein 16, AtMYB16, e-value 1×10^−73^). Similarity with R2R3-MYB transcription factors known to control epidermal cell differentiation [40], [59] (see below) pointed to this gene as a likely candidate for the peach/nectarine trait [54]. This second variant was also detected by Pindel software in C×A F_1_ and ‘Quetta’ samples only, as a large insertion compared to the reference sequence. In particular, this large insertion at position 15,898,458 was supported in Pindel by a total of 31 reads, 17 overhanging on the left side of the insertion (10 in C×A F_1_ and 7 in ‘Quetta’) and 14 on its right side (5 in C×A F_1_ and 9 in ‘Quetta’). The presence of this insertion in the nectarine allele is also supported by analysis of ‘Quetta’ resequencing data using CLC Genomic Workbench. Within the considered mapping interval (from 15,853,006 to 16,488,104) a total of 94,789 reads (9.15 million nucleotides) were aligned against the reference genome sequence, 61% of which in pairs and the remaining as single reads due to unexpected mapping distances, mate inversion, unmapping or mapping in other contigs. In agreement with Pindel results, the third exon of the *ppa023143m* gene showed a dramatic reduction of paired-end distances and an increase of single reads at position 15,898,458 (Figure 2), compatible with a large insertion in ‘Quetta’ compared to the ‘Lovell’ reference sequence. Due to this insertion only single reads could align in the region; the software reports the lack of paired reads assigning the value zero to the paired-end distance and increasing to 100% the percentage of single reads (Figure 2).

### Validation and reconstruction of a long insertion in exon 3 of gene ppa023143m

**Figure 3:**
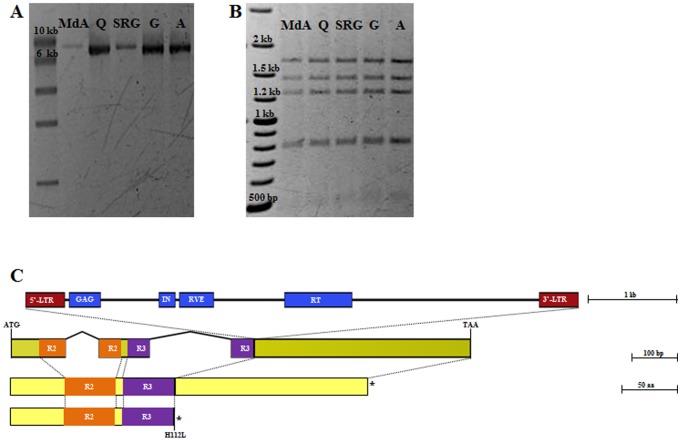
Variant discovery in *PpeMYB25* (annotation refinement of *ppa023143m*). Five nectarine genotypes (‘Madonna di Agosto’, MdA; ‘Quetta’, Q; ‘Stark Red Gold’, SRG; ‘Goldmine’, G; ‘Ambra’, A) were analyzed to confirm the presence of the insertion within exon 3 of *PpeMYB25*. (A) Long-range amplification products reveal for all the accessions a fragment of about 7 kb (compared to 960 bp expected from the reference genome). (B) Double digestion results of the long-range PCR products show the same pattern for all the genotypes. (C) Position and structure of the Ty-*copia* retrotransposon deduced by the by the NGS analysis of ‘Quetta’ long-range amplicon. The insertion results in a truncated version of the R2R3-MYB protein.

The physical presence of the long insertion within exon 3 of gene *ppa023143m* was confirmed by long-range PCR using a primer pair designed on intron 2 (Seq16F) and exon 3 (Seq4R) flanking the insertion site. An amplification product of about 7 kb was obtained in five nectarines, ‘Madonna di Agosto’, ‘Quetta’, ‘Stark Red Gold’, ‘Goldmine’ and ‘Ambra’ (Figure 3). In contrast, in a peach genotype (‘Contender’) the same primer pair gave an amplification product of 960 bp (data not shown). ‘Quetta’ and ‘Goldmine’ are two donors of the trait in modern breeding and ‘Stark Red Gold’ is known to carry the nectarine allele of ‘Lippiat’, the third donor of the trait. ‘Madonna di Agosto’ belongs to a group of landraces not directly related to modern breeding germplasm [12], [13]. The double digestion of the five amplicons, with *EcoR*I/*Hind*III, shows the same restriction pattern for all the accessions (Figure 3) suggesting that a unique mutational event gave origin to the nectarine trait present in the modern nectarine germplasm as well as in the local southern Italian ecotypes.

The amplicon obtained in ‘Quetta’ was sequenced by Next Generation Sequencing (NGS). Following filtering and assembly 90% of the reads were collected in three major contigs. Contig_1 (GenBank accession number KJ150676), formed by a consensus sequence of 5,836 bp, was mapped on scaffold 3 of peach genome v1.0 and showed a perfect match for 5,713 bp (scaffold_3:13,409,926.13,415,638) corresponding to the predicted LTR_1684 region (http://services.appliedgenomics.org/gbrowse/prunus_public/). The missing 123 bp that do not align on scaffold 3 showed a perfect match on scaffold 5 (scaffold_5:15,898,458.15,898,581), confirming that this contig represents an insertion in the third exon of predicted gene *ppa023143m*. When the LTR_1684 region, found in correspondence of Contig_1 alignment on scaffold 3, was submitted to CENSOR [60] (Release 18.01, 16 Jan 2013) high similarity was found to a 6,033 bp annotated Ty1-*copia* retrotransposon (Copia-24_FV-I) from strawberry (*Fragaria vesca*). A conserved protein domain analysis on the complete sequence of Contig_1 also revealed the presence of different domains: UBN2 (gag-polypeptide of LTR copia-type) from position 1,354 to 1,689 of Contig_1, GAG-pre-integrase domain from position 2,329 to 2,511, RVE (Integrase core domain) from position 2,551 to 2,928 and RVT2 (Reverse transcriptase domain) from position 3,694 to 4,431. The four domains identified showed a high prediction confidence, with E-values of 3.8e-16, 4.3e-13, 6.8e-28 and 1.6e-98 respectively. All these predicted domains are typical of retrotransposable elements. The remaining two contigs, Contig_2 and Contig_3, 300 and 876 bp respectively, were also mapped on the peach genome. Both were split on scaffold 5 (scaffold_5:15,898,340.15,898,361; scaffold_5: 15,898,458. 15,899,262) in exon 3 of predicted gene *ppa023143m* and on scaffold 3 in the same LTR_1684 region of Contig_1. Thus, these contigs span the point of insertion of the Ty-*copia* retroelement within exon 3 of the gene. Flanking this insertion point, we found a characteristic Target Site Duplication (TSD) (AC)_3_ [61] produced by the retroelement upon insertion into a new site. Together these results confirm the insertion of a Ty1-*copia* retrotransposon in the ‘Quetta’ allele of gene *ppa023143m*.

A BLASTN search of the retrotransposon sequence of Contig_1 against the peach reference genome returned 5 highly similar hits (from 87.1% to 100% sequence identity), two on chromosome 3, and one each on chromosomes 4, 7 and 8. All five hits were about 6 kb long and were precisely delimited by highly similar (from 97.7% to 100.0%) LTR sequences, each flanked by characteristic Target Site Duplications, thus confirming the existence of other copies of this LTR-retroelement in the reference peach genome. In particular LTRs found in the ‘Quetta’ allele of *ppa023143m* are identical to those present in scaffold 3 (scaffold_3:14,488,093.14,488,522) and scaffold 4 (scaffold_4:27,503,652.27,504,081). These data are consistent with previous analyses showing that 12.6% of LTR-retrotransposons have identical LTRs, indicating recent and ongoing retrotransposition activity in the peach genome [8].

Transposable elements (TEs) are known to cause many kinds of genetic variations in plants and played an important role in plant evolution and domestication [62]. In a survey of allelic variants at 60 genes involved in crop domestication and diversification, 15% were caused by TE insertions [63]. For example, an LTR insertion in a regulatory region of the *teosinte branched1 (tb1)* gene resulted in overexpression of the gene, causing the conversion from highly branched wild teosinte to the single culm architecture of domesticated maize [64], [65]. However, the most common effect of TE insertion is the loss of gene function [57] and recessive TE-induced mutations have played an important role in plant domestication [62]; examples include “sticky” foxtail millet [66], Mendel's wrinkled peas [67], seedless apple [68], fruit color in grape [69]–[71] and peach flesh [72], [73]. In some cases, multiple independent mutational events were selected as demonstrated by insertion of TEs in the *waxy* locus resulting in the “sticky” phenotype in foxtail millet [66]. Another example is the yellow peach phenotype, which is associated with three different mutational events (an LTR insertion, a SNP and a frameshift mutation at a microsatellite locus) that occurred independently in a carotenoid cleavage dioxygenase gene directly involved in pigment degradation [72], [73]. In contrast with the situation for “sticky” foxtail millet [66] and peach color [72], [73] our results suggest that a unique mutational event has originated the nectarine phenotype, i.e. the loss of trichomes in peach fruit. The occurrence of unique mutations affecting single genes selected by humans during domestication and diversification of crop species is not rare. In a recent review of 60 such genes, 26 display a unique mutational event selected and spread by humans [63].

### Gene ppa023143m encodes an R2R3-MYB transcription factor putatively involved in trichome formation

**Figure 4:**
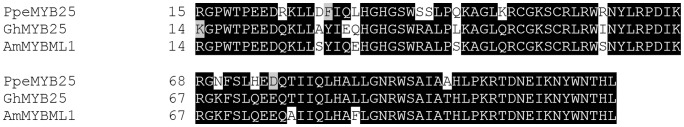
Aminoacid alignment of the R2 and R3 MYB repeat sequences. MYB domains (pfam00249) of peach PpeMYB25, cotton GhMYB25 (ACJ07153.1, [39]) and *Antirrhinum* AmMYBML1 (CAB433991.1, [54]) were aligned using the Muscle on line tool at EBI (http://www.ebi.ac.uk/Tools/msa/muscle/). Graphic display of the alignment was obtained using BoxShade (http://www.ch.embnet.org/software/BOX_form.html). Black shaded residues are identical, grey shaded residues are similar. Coordinates in the protein sequences are indicated.

Careful inspection of predicted gene *ppa023143m* led us to reannotate the coding sequence (CDS) extending exon 3 compared to the Peach v1.0 annotation [8]. The reannotated CDS is predicted to encode a peptide of 330 aminoacids showing similarity to the R2R3-MYB transcription factors GhMYB25 from allotetraploid cotton *Gossypium hirsutum* (58.4% similarity) [40] and MIXTA-like1 from *Antirrhinum* (AmMYBML1, 55.3% similarity) [59] (Figure 4). In eukaryotes, MYB factors represent one of the largest and most functionally diverse gene families, which dramatically expanded in plants [74]–[77] playing a central role in a variety of processes from plant development to responses to biotic and abiotic stresses [74], [78], [79]. MYB family members share a highly conserved DNA binding domain (the MYB domain) usually composed of up to three amino acid repeats (R1, R2, R3) [80]. *GhMYB25* and *AmMYBML1* belong to R2R3-MYB sub-group 9 [40], [77] along with other genes involved in regulating petal epidermal cell shape [81]. *AmMYBML1* plays a role in trichome differentiation in the corolla tube of the *Antirrhinum* flower [59]. *GhMYB25*, normally expressed at the time of fiber initiation in the outer integument of ovules, is differentially expressed between fibreless mutants and normal lined cotton [40], [82], [83] and its altered expression affects seed, fiber and trichome development in transgenic cotton [40]. Considering the known role of these homologues in trichome development, *ppa023143m* is a strong candidate for the peach/nectarine phenotype and was named *PpeMYB25*. The insertion of the Ty1-*copia* retrotransposon in exon 3 of the *PpeMYB25* gene introduces an H112L substitution and a premature stop codon (TAA), resulting in a peptide of 112 aminoacids precisely truncated at the C-terminal end of the R3 MYB domain. GhMYB25 and AmMYBML1 share the distinctive C-terminal motif of R2R3-MYB sub-group 9 [40], [59], [76] along with *PpeMYB25* and other genes involved in regulating epidermal cell shape [81]. Analysis of *Antirrhinum* R2R3-MYB genes from subgroup 9 indicated that the C-terminal domain folds as an amphipathic α-helix with putative transactivation ability [84]. An insertional mutant in the MIXTA gene, resulting in loss of this C-terminal region has been shown to cause recessive phenotypic alteration in epidermal cell differentiation [85]. The recessiveness of the nectarine trait indicates that it corresponds to a loss-of-function mutation. According to this reasoning and by analogy with observations in cotton, we propose that the observed insertion in the *PpeMYB25* gene results in a non-functional form of the MYB transcription factor that normally promotes trichome formation in fuzzy peaches. If this were correct, all nectarines should be non-functional homozygous mutants at this gene.

**Figure 5:**
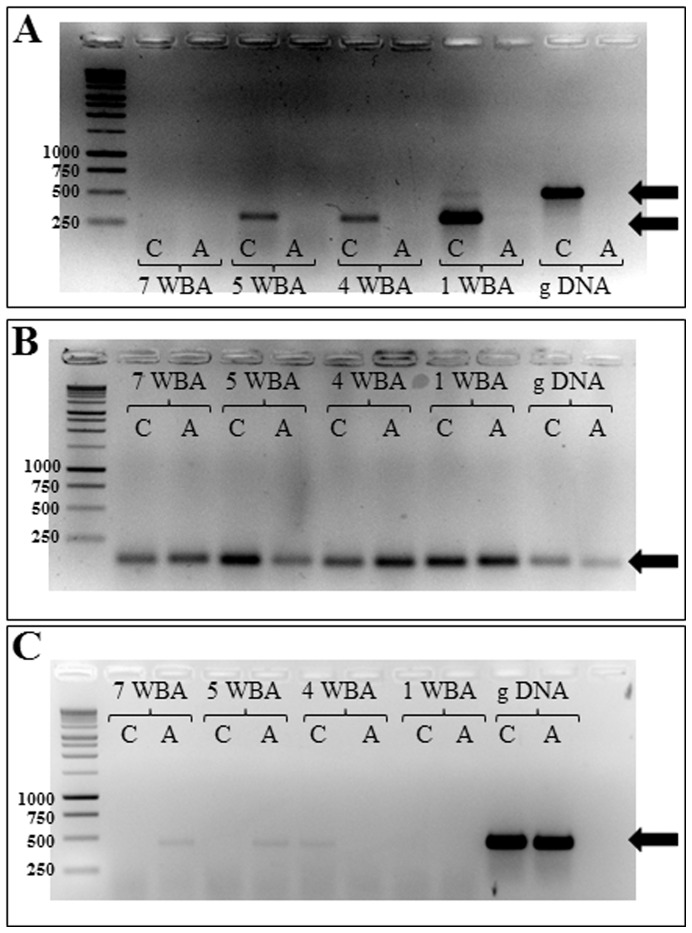
Expression analysis of *PpeMYB25* in peach and nectarine flower buds. (A) The expression patterns of the R2R3-MYB gene were evaluated in ‘Contender’ [C] and ‘Ambra’ [A] buds at seven, five, four and one week before anthesis (WBA). Genomic DNA of the two cultivars was also tested as a control. The same samples were analyzed for expression of *RPII* as standard (B) and checked for DNA contamination (C).

In order to evaluate the timing of *PpeMYB25* transcript expression with respect to trichome development, RT-PCR analyses were performed on ‘Contender’ and ‘Ambra’ floral buds sampled at seven, five, four and one week before anthesis. A forward primer designed on exon 2 was used in combination with a reverse primer on exon 3 (downstream of the LTR insertion) in order to evaluate the expression profiles of the gene. Expression was evident in ‘Contender’ from five weeks before anthesis, just before trichomes begin to appear [26] and continued through to one week before anthesis accompanying trichome differentiation (Figure 5). In contrast, the expression of *PpeMYB25* was never visible in ‘Ambra’ floral buds, consistent with the presence and the position of the large insertion (Figure 5). Together sequence and expression analyses support the proposed involvement of *PpeMYB25* gene in promoting trichome differentiation. Mutational events in regulatory genes have played a major role during crop domestication and breeding; in a recent overeview of domestication and diversification genes, 37 out of 60 (∼62%) encoded transcription factors [63].

### Association study and origin of the nectarine trait in peach germplasm

**Figure 6:**
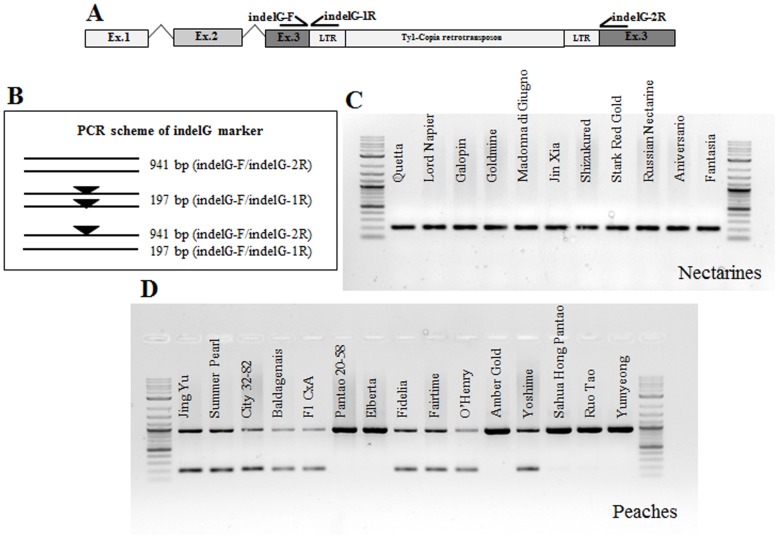
Functional Marker indelG. A marker assay was developed based on sequence information on the *PpeMYB25* gene and the Ty1*-copia* insertion. Three primers were designed to discriminate peach and nectarine genotypes (A, B). A panel of nectarines including the putative donors of the trait, show a unique fragment of about 200 bp (C). A set of peaches, of diverse pedigree and origins (Table 1) (D), shows homozygous or heterozygous patterns.

The genotype at the LTR retrotransposon insertion in gene *PpeMYB25* was assessed in the CxA F_2_ population as well as in a panel of 95 peach and nectarine accessions by means of a functional marker based on a three primers PCR assay (indelG) (Figure 6). As expected, the marker co-segregated with the *G* locus in the CxA F_2_ progeny (Figure 1), with all the nectarines displaying a unique fragment of 197 bp. Similarly, all nectarines in the germplasm panel were characterized by a fragment of the same length (Figure 6). Peach accessions fell into two categories: those homozygous for the reference allele (941 bp) and those heterozygous (197 bp, 941 bp). Taking into account pedigree information [15], [86], we confirmed all the known heterozygotes (‘Autumnglo’, ‘Fairtime’, ‘Fidelia’, ‘Jing Yu’, ‘O’Henry’, ‘Summer Pearl’ and CxA F_1_, Table 1) and we also detected three previously unknown heterozygous genotypes (‘Baldagenais’, ‘City 32–82’ and ‘Yoshihime’) carrying the nectarine allele originated from the LTR insertion. The complete association between the indelG marker and the trait confirms the presence of the Ty1*-copia* retrotransposon within exon 3 of the *PpeMYB25* gene in all the nectarines analyzed.

Modern western nectarines trace back to three founders discovered at the beginning of the last century (‘Quetta’ in Pakistan, and ‘Lippiatt’ and ‘Goldmine’ in New Zealand). The founders and their modern descendants show the presence of the retrotransposon insertion. The insertion is also present in Southern Italian traditional landraces (‘Madonna di Giugno’ and ‘Madonna di Agosto’) cultivated since the 16^th^ century [12]. In addition to these genotypes, we confirmed the presence of the retrotransposon in several non-related accessions including old European landraces (‘Lord Napier’ and ‘Galopin’) [15], modern Asian cultivars (Chinese and Japanese) and different nectarine cultivars of unknown pedigree (from Italy, East Europe, USA, Mexico and South America). The modern Asian nectarines analyzed in this study (‘Chiyodared’, ‘Shizukured’ and ‘Jin Xia’) have two old European landraces, ‘Lord Napier’ and ‘Precoce di Croncels’, as donors of the nectarine trait. No traditional nectarine landraces have been reported in China [14] and all modern Chinese nectarines inherited the trait from western germplasm [17].

Together these results indicate that all known nectarine germplasm derives from a unique mutational event in *PpeMYB25* selected and spread by humans during peach dissemination and breeding.

## Conclusions

Nectarines play an important role in the peach industry. In the present study, using a candidate gene approach coupled with fine mapping and NGS-based variant discovery, we provide strong evidence that the transcription factor gene *PpeMYB25* acts as a positive regulator of trichome formation in peach fruit. The insertion of a Ty1-*copia* retrotransposon within the third exon of *PpeMYB25* was identified as the putative cause of a loss-of-function mutation underlying the nectarine phenotype, further supporting the importance of transposition in plant genome evolution and phenotypic variability in domesticated crops. Finally, the development of a functional marker, indelG, provides an efficient diagnostic tool for the early selection of the peach/nectarine trait in marker assisted breeding (MAB).
