# Correction: A Unique Mutation in a MYB Gene Cosegregates with the Nectarine Phenotype in Peach

**DOI:** 10.1371/journal.pone.0112032

**Published:** 2014-10-27

**Authors:** The PLOS ONE Staff

In Figure 3, the stop codon is labeled incorrectly. The authors have provided a corrected version here.

**Figure 3:**
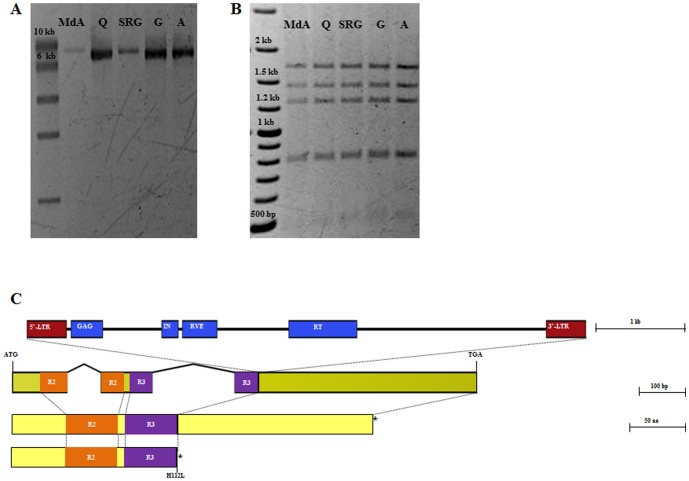
Variant discovery in PpeMYB25 (annotation refinement of ppa023143m). Five nectarine genotypes (‘Madonna di Agosto’, MdA; ‘Quetta’, Q; ‘Stark Red Gold’, SRG; ‘Goldmine’, G; ‘Ambra’, A) were analyzed to confirm the presence of the insertion within exon 3 of PpeMYB25. (A) Long-range amplification products reveal for all the accessions a fragment of about 7 kb (compared to 960 bp expected from the reference genome). (B) Double digestion results of the long-range PCR products show the same pattern for all the genotypes. (C) Position and structure of the Ty-copia retrotransposon deduced by the by the NGS analysis of ‘Quetta’ long-range amplicon. The insertion results in a truncated version of the R2R3-MYB protein.

In Figure 6, the positions of the primers, indelG_F and indelG_2R are incorrect. The authors have provided a corrected version here.

**Figure 6:**
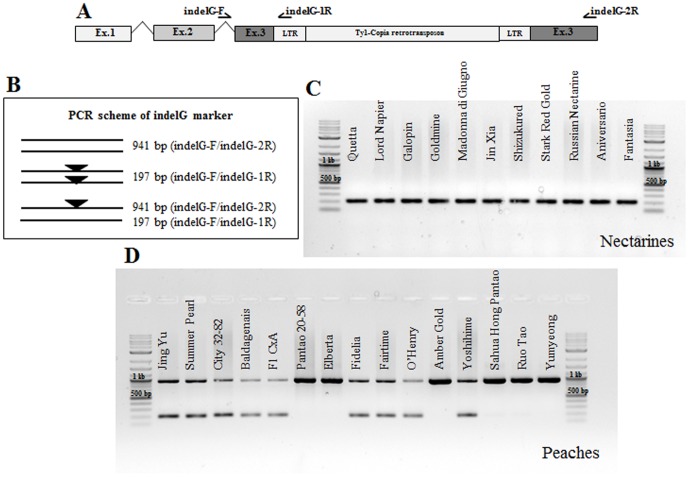
Functional Marker indelG. A marker assay was developed based on sequence information on the PpeMYB25 gene and the Ty1-copia insertion. Three primers were designed to discriminate peach and nectarine genotypes (A, B). A panel of nectarines including the putative donors of the trait, show a unique fragment of about 200 bp (C). A set of peaches, of diverse pedigree and origins (Table 1) (D), shows homozygous or heterozygous patterns.
